# Polydim-I antimicrobial activity against MDR bacteria and its model membrane interaction

**DOI:** 10.1371/journal.pone.0178785

**Published:** 2017-06-01

**Authors:** Marisa Rangel, Fabíola Fernandes dos Santos Castro, Lilian Daiene Mota-Lima, Patricia Bianca Clissa, Danubia Batista Martins, Marcia Perez dos Santos Cabrera, Marcia Renata Mortari

**Affiliations:** 1 Immunopathology Laboratory, Butantan Institute, Sao Paulo-SP, Brazil; 2 Laboratory of Neuropharmacology, Department of Physiological Sciences, Institute of Biological Sciences, University of Brasília, Brasília-DF, Brazil; 3 Departamento de Física, Universidade Estadual Paulista, UNESP, São José do Rio Preto, SP, Brazil; 4 Departamento de Química e Ciências Ambientais, Universidade Estadual Paulista, UNESP, São José do Rio Preto, SP, Brazil; nanyang technological university, SINGAPORE

## Abstract

The rapid spread of multi-drug resistant pathogens represents a serious threat to public health, considering factors such as high mortality rates, treatment restrictions and high prevalence of multi-drug resistant bacteria in the hospital environment. Antimicrobial peptides (AMPs) may exhibit powerful antimicrobial activity against different and diverse microorganisms, also presenting the advantage of absence or low toxicity towards animal cells. In this study, the evaluation of the antimicrobial activity against multi-drug resistant bacteria of a recently described AMP from wasp, Polydim-I, was performed. Polydim-I presented activity against standard strains (non-carriers of multi-resistant genes) that are susceptible to commercial antimicrobials, and also against multi-drug resistant strains at concentrations bellow 1μg/ml (0.41 μM). This is a rather low concentration among those reported for AMPs. At this concentration we found out that Polydim-I inhibits almost 100% of the tested pathogens growth, while with the ATCC strains the minimum inhibitory concentration (MIC_100_) is 400 times higher. Also, in relation to *in vitro* activity of conventional drugs against multi-drug resistant bacteria strains, Polydim-I is almost 10 times more efficient and with broader spectrum. Cationic AMPs are known as multi-target compounds and specially for targeting the phospholipid matrix of bacterial membranes. Exploring the interactions of Polydim-I with lipid bilayers, we have confirmed that this interaction is involved in the mechanism of action. Circular dichroism experiments showed that Polydim-I undergoes a conformational transition from random coil to a mostly helical conformation in the presence of membrane mimetic environments. Zeta potential measurements confirmed the binding and partial charge neutralization of anionic asolectin vesicles, and also suggested a possible aggregation of peptide molecules. FTIR experiments confirmed that some peptide aggregation occurs, which is minimized in the presence of strongly anionic micelles of sodium dodecyl sulfate. Also, Polydim-I induced channel-like structures formation to asolectin lipid bilayers, as demonstrated in the electrophysiology experiments. We suggest that cationic Polydim-I targets the membrane lipids due to electrostatic attraction, partially accumulates, neutralizing the opposite charges and induces pore formation. Similar mechanism of action has already been suggested for other peptides from wasp venoms, especially mastoparans.

## 1 Introduction

Since the beginning of the clinical use of antimicrobial agents, strains of multi-resistant bacteria have emerged. The fast dissemination of these multi-resistant pathogens represents a threat to public health, considering the high mortality rates, treatment restrictions and the prevalence in hospitals of bacteria resistant to antimicrobials [1]. Recently, an increase in the number of infections has been induced by a group of pathogens named by the acronym ESKAPE: *Enterococcus faecium*, *Staphylococcus aureus*, *Klebsiella pneumonia*, *Acinobacter baumanii*, *Pseudomonas aeruginosa* and *Enterobacter* sp. The term ESKAPE is a reference to the difficulties to eliminate infections caused by these pathogens due to several mechanisms of escape they possess, and to the urgency in the discovery of new antimicrobial drugs, capable of eliminating infections they induce [2]. Although these bacteria do not share the same mechanisms of resistance induction, they share an increasing prevalence due to the selective pressure exerted by public policies (or their absence) for antibiotics use, especially in the Intensive Care Units (ICUs) [3]. Antimicrobial resistant infections are responsible for approximately 50,000 deaths per year only in USA and Europe. Worldwide, the number of deaths attributed to antimicrobial resistant infection is estimated to be above 700,000/year. If no action is taken to control the growing rates of infection induced by such pathogens, about 10 million lives can be taken per year by the year of 2050 [4].

AMPs are ubiquitous in living organisms, and are in general, molecules of the innate immune system, which exert potent antimicrobial activity against a wide range of microorganisms, associated to the absence or reduced toxic effects towards mammalian cells [5]. This reduced toxicity is attributed to biochemical and structural differences between cell membranes of microorganisms and mammals. The lipid bilayer of the bacteria membrane is composed of negatively charged phospholipids, facing outside the cell. In mammalian cells, however, zwitterionic phospholipids facing the extracellular compartment form the membrane, which also includes cholesterol, that in animal cells decreases the lytic effects of AMPs, and is absent in bacterial membranes [6,7].

Typical AMPs consist of 10 to 50 amino acid residues with positive net charge. Folded, they may acquire mostly four types of secondary structures: α-helix, β-sheet, combined α-helix and β-sheet, and extended. In general, the mechanism of action of AMPs is either directed by electrostatic interaction towards the microbe membrane, followed by pore formation or membrane rupture, or penetrate the bacterial cytosol by passing through an outer membrane protein, or by channels formed by the AMPs, or by a flip-flop mechanism, and exert antimicrobial effect by inhibiting protein or DNA synthesis [8,9,10]; alternatively, AMPs may modulate the host immunity system [11].

Two main general mechanisms of action for peptide disruption of membrane were purposed: pore formation through the lipid bilayer or the carpet mechanism that could either cause intermittent leaky defects on the membrane or its lysis as a detergent. Further detailed description of the mechanisms of pore formation and bacterial membrane destabilization by AMPs can be revised on previous articles [12–19].

The development of microbial resistance against AMPs is rare [20]. Although some pathogens are capable of expressing mechanisms to avoid the AMP target and bypass the host defenses [15,21], AMPs represent one of the most promising strategies in the fight against infections and resistance to antimicrobial drugs. There is a major interest, regarding public health, in the development of new antimicrobial classes, since in the last four decades just three were introduced as medicines (lipopeptides, oxazolidinones and streptogramins) [22,23]. Besides being rare that bacteria develop mechanisms of resistance against antimicrobial drugs which target membrane lipids, these antimicrobials are active against slow-growing or dormant bacteria and on biofilms [24]. In addition to the already proven antibacterial activity of AMPs individually administrated [25], there is the possibility of combined use with conventional drugs [22,26,27].

Wasp venoms are composed by substances developed through an elaborated evolution process, and effectively used to self-defense, immobilization or capture of the prey. The venom of social wasps contains a variety of bioactive compounds, with a number of pharmacological activities, including antimicrobial activity [28,29]. Several bioactive peptides were isolated from wasp venoms, and the most studied class is the one of the mastoparans. Mastoparan peptides are rich in lysine residues and play an important role in the stimulation and release of biogenic amines such as mast cells histamine. Furthermore, mastoparans present potent antimicrobial activity [30].

Polydim-I is a peptide recently isolated from the venom of the Neotropical wasp *Polybia dimorpha* that is active against *Mycobacterium abscessus* subsp. *massaliense* infections *in vitro* and *in vivo* [31]. The peptide has 22 amino acid residues. Although presenting an amphipathic structure due to the presence of hydrophobic amino acid residues (methionine, leucine, valine, and proline) intercalated with negatively charged ones, as other wasp AMPs do, Polydim-I also presented particular intrinsic characteristics, which made it be considered a new class of AMP. Morphological analysis of *M*. *abscessus* subsp. *massiliense* cell surface indicated that Polydim-I activity could be related to cell membrane integrity disruption. Besides the efficacy demonstrated on the reduction of *M*. *abscessus* subsp. *massaliense* infections in macrophages and mice models, Polydim-I also showed selective toxicity. Toxicity in macrophages was only 10% when they were treated with concentrations 15–16 times higher (121.6 μg/mL) than the concentration used to reduce in 50% the infection in the same cells (7.6 μg/mL). Furthermore, the haemolytic activity against human erythrocytes (only 2.5% at 121.6 μg/mL) can be considered insignificant [31].

The present study aimed at expanding the characterization of the antimicrobial potential of Polydim-I, through the determination of its minimum inhibitory concentration (MIC) against ATCC sensitive bacteria strains and ESKAPE pathogens. Additionally, the detection of defects on resistant bacteria cell membrane by SEM, indicating pore-formation as a possible mechanism of action [31] led us to confirm the possibility of pore formation and to evaluate by other methods its structural features and mechanism of interaction with the lipid matrix that could make it a candidate to drug development studies.

## 2 Materials and methods

### 2.1 Peptide synthesis

Polydim-I (AVAGEKLWLLPHLLKMLLTPTP-_NH2_; m/z 2441.7 [M+H]^+^) was synthesized using Fmoc chemistry by AminoTech P & D (Sao Paulo, Brazil). The correct sequence and purity (>99%) were evaluated by HPLC and mass spectrometry.

### 2.2 Antibacterial activity

ATCC (American Type Culture Collection) strains were acquired from BioMerieux as a part of the kits Lyfocults® of the quality control of the equipment Vitek2® of the same company. The multi-resistant strains were provided by the bacterial library of the Microbiology Laboratory of the Centro Universitário de Brasília (UNICEUB). The microorganisms used were: *Staphylococcus aureus* ATCC2 5923, *Staphylococcus aureus* methicillin resistant (MRSA); *Enterococcus faecalis* ATCC 29212; *Enterococcus faecalis* vancomycin resistant. *Escherichia coli* ATCC 25922; *Pseudomonas aeruginosa* ATCC27853; *Acinetobacter baumannii* ATCC 19606; *Klebsiella pneumonia* carbapenemase (KPC)-producing; extended spectrum betalactamase (ESBL)-producing *Escherichia coli*; metallo-β-lactamases producing *Pseudomonas aeruginosa* resistant to carbapenems.

MICs for Polydim-I against bacteria strains were determined by 2-fold serial broth micro dilution in Müeller-Hinton broth (Difco™, BD, USA) in 96-well plates. Each well received 90 μl of peptide solutions plus 90 μl of the broth. In each well 20 μl of bacterial suspension was added, resulting in a final volume of 200 μl at the concentration of 10^5^ CFU/ml. Controls were: DMSO 2.5% (vehicle) with and without bacterial suspension, Polymyxin B, Meropenem, and Vancomycin (all from Sigma-Aldrich Co., USA). Following inoculation, the plates were incubated at 37°C for 18 h before measuring the turbidity of the cultures at 595 nm in an ELISA reader (MultiSkan FC®, Thermo Scientific, USA) to assess bacterial growth. The results were expressed as inhibition percentage of optical density (OD) against a control in the absence of the peptide or the control antibiotics. MIC_50_ was calculated through non-linear regression of the dose-response growth inhibition curves for Polydim-I against the ATCC strains, and MIC_100_ was the concentration that completely inhibited bacterial growth.

### 2.3 Large unilamellar vesicles preparation for spectroscopy experiments (LUV)

Multi-layered vesicles (MLVs) were prepared from films of asolectin (Sigma-Aldrich Co., USA) deposited on the bottom of round flasks after solvent (chloroform) evaporation under N_2_ flow, followed by vacuum drying for at least 3 h. Films were hydrated with buffer (citrate/phosphate pH 5.5 containing 150 mM NaF), at 40^°^C, to reach a final lipid concentration of 10 mg/mL and vortex mixed. MLVs were submitted to an extrusion process through polycarbonate membranes (Whatman® Nuclepore Track-etch Membrane, Sigma-Aldrich Co., USA) in two steps: first, through 400 nm membranes (6 times), then through two stacked 100 nm membranes, using an Avanti mini-extruder (Avanti Polar Lipids Inc., USA), at 40^°^C, rendering LUVs. For ATR-FTIR experiments vesicles were prepared in deuterated buffer and left incubating for 2h prior to spectra acquisition.

Peptide samples were prepared by diluting the stock solution (in D_2_O for IR experiments) with buffer/deuterated buffer, or with buffer containing either 40% 2,2,2-trifluoroethanol (or 2,2,2-trifluoroethanol-d3, TFE), or 8 mM sodium dodecyl sulfate (SDS) or asolectin (AZO) LUVs, at 100 and 250 μg/mL.

### 2.4 Circular dichroism (CD) spectroscopy

CD spectra were obtained in the absence and in the presence of peptide at 20 μM concentration, at 25°C, recorded from 260 to 202 or 190 nm (depending on signal-to-noise ratio) with a Jasco-710 spectropolarimeter (JASCO International Co. Ltd., Tokyo, Japan) Spectra were acquired using 0.5 cm path length cell, averaged over ten scans, at a scan speed of 50 nm/min, bandwidth of 1.0 nm, 0.5 s response and 0.2 nm resolution. Baseline correction was applied. The mean-residue ellipticity, [Θ] (deg cm^2^/dmol), was calculated with the relationship [Θ] = 100θ/(lcn), where θ (mdeg), is the observed ellipticity, ‘l’ the path length in cm, ‘*c*’ (mM) the peptide concentration, and ‘*n*’ the number of peptidic bonds. Spectra were fitted with the software CDPro (configured with CONTIN/LL and using the reference set SMP56).

### 2.5 Attenuated Total Reflectance-Fourier-Transform Infrared Spectroscopy (ATR-FTIR)

FTIR spectra were recorded in the range from 1600 to 1700 cm^-1^ with the spectrophotometer Spectrum Two (Perkin Elmer, Beaconsfield, UK), fitted with ATR device. Aliquots of 7 μL of each sample were deposited on top of the ATR crystal device. To obtain the difference spectra, samples were analyzed in the absence and in the presence of peptide at 1 mM concentration. For each spectrum 20 scans were collected with resolution of 2 cm^-1^. The background was the clean crystal. To assign the wave numbers of the component bands in the spectra, baseline correction was applied, overlapping bands were resolved and the second-derivative spectra were smoothed by 13-data point Savitsky-Golay.

The ATR-FTIR spectra were analyzed to give the estimates of the relative contents of the different secondary structure elements included in the whole area of the amide I band based on the fraction of total area under the peak. The deconvolution of the amide I region considered the following peaks and assignments to the secondary structure elements: between 1620–1625 cm^-1^, aggregated β-sheet; 1625–1640 cm^-1^ β-sheet, from 1645–1655 cm^-1^, α-helix; 1656–1670 cm^-1^, distorted helix; 1670–1680 cm^-1^, turns [32,33].

### 2.6 Size and zeta potential determinations

The Zetasizer Nano ZS (Malvern Instruments, Worcestershire, U.K.) was used to determine size and the zeta potential of AZO LUVs in the absence and in the presence of the peptide. Peptide solutions were prepared in buffer, pH 5.5, in plastic vials and an aliquot of the vesicle suspension was added to a final concentration of 250 μg/mL. After 30 minutes equilibration at 25^°^C the preparation was transferred to a disposable cuvette for size evaluation. Then, the same preparation was transferred to a DTS1070 cell (Malvern Instruments, England) for zeta potential measurement. Values of viscosity and refractive index were 0.8872 cP and 1.330, respectively.

### 2.7 Giant unilamellar vesicles (GUVs), bilayer formation and electrophysiology

The giant unilamellar vesicles were prepared from asolectin (Sigma-Aldrich Co., USA) and formed by electroswelling, using the device Vesicle Prep Pro (Nanion Technologies GmbH, Germany) following the protocol described by Rangel et al., 2011 [34]. Lipid bilayers were formed from GUVs after added to the borosilicate glass chips NPC-1 aperture. Electrical measurements were performed with the automated Patch-Clamp device Port-a-Patch® (Nanion Technologies GmbH, Germany) as previously described [34].

## 3 Results

### 3.1 Antibacterial activity

The growth inhibition results of Polydim-I against ATCC bacteria strains are summarized in Table 1. The peptide presented low MIC against *Staphylococcus aureus*, with a MIC_50_ of 4.1μg/ml. Against *Pseudomonas aeruginosa*, however, concentrations higher than 400μg/ml were tested, and yet the MIC_50_ and MIC_100_ could not be precisely calculated. Peptide concentrations that inhibited 50% of the growth of *Enterococcus faecalis*, *Acinetobacter calcoaceticus-baumannii* and *Escherichia coli* were, respectively: 73.2, 84.0 and 50.7 μg/ml. *E*. *coli* was the Gram-negative bacteria more susceptible to the peptide treatment. MIC_100_ values were equal or superior to 400 μg/ml for all tested ATCC bacteria. As a control of inhibition of the bacterial growth, commercial antimicrobial drugs were tested against the same standard strains (Table 2).

**Table 1:** Polydim-I activity against ATCC bacteria strains (in μg/ml, with 95% confidence limits for MIC_50_ presented in parentheses). MICs were determined by 2-fold serial micro dilution in Müeller-Hinton broth in 96-well plates. Each well received 90 μl of peptide plus 90 μl of broth. In each well 20 μl of bacterial suspension was added, resulting in a final volume of 200 μl at the concentration of 10^5^ CFU/ml. The plates were incubated (37°C, 18 h) and turbidity was measured at 595 nm to assess bacterial growth. The results were expressed as inhibition percentage of optical density (OD) against a control in the absence of the peptide. Three independent experiments were performed in triplicate.

	**ATCC bacteria strains**				
**Polydim-I(μg/ml)**	*S*. *aureus*	*E*. *faecalis*	*E*. *coli*	*P*. *aeruginosa*	*A*. *calcoaceticcus*
**MIC_50_**	4.1 (1.5–11.5)	73.2 (56–95.6)	50.7 (33–78)	> 400	84 (65–109)
**MIC_100_**	400	400	>400	>400	>400

**Table 2:** Inhibition of ATCC bacteria strains by commercial antibiotics, in percentage ± SEM. Each well received 90 μl of antibiotic solutions plus 90 μl of broth. In each well 20 μl of bacterial suspension was added, resulting in a final volume of 200 μl at the concentration of 10^5^ CFU/ml. The plates were incubated (37°C, 18 h) and turbidity was measured at 595 nm to assess bacterial growth. The results were expressed as inhibition percentage of optical density (OD) against a control in the absence of the control antibiotics. Three independent experiments were performed in triplicate.

		**ATCC bacteria strains**				
**Drugs**	Concentration (μg/ml)	*S*. *aureus*	*E*. *faecalis*	*E*. *coli*	*P*. *aeruginosa*	*A*. *calcoaceticcus*
**Vancomycin**	10	90.9 ± 1.3	74.6 ± 2.3	NT	NT	NT
	100	95.2 ± 0.7	89.7 ± 0.4	NT	NT	NT
**Meropenem**	10	NT	NT	97.5 ± 1.7	94.9 ± 4.8	99.0 ± 0.6
	100	NT	NT	96.1 ± 0.05	96.7 ± 5.3	100
**Polymyxin B**	10	NT	NT	87.6 ± 6.7	98.9 ± 0.1	92.5 ± 1.75
	100	NT	NT	85.3 ± 5.3	97.9 ± 2.0	99.8 ± 0.1

**NT:** non tested because there is no indication for use

To confirm the standard response of the strains used in this study, commercial drugs with reduced or absent activity against bacteria expressing resistance genes were tested. Thus, vancomycin was tested against VRE strains, and meropenen against *Pseudomonas aeruginosa* and *Acinetobacter* sp. In parallel, antimicrobial drugs indicated for the treatment of infections caused by multi-resistant microorganisms were also tested: vancomycin against MRSA strains and e polymyxin B on *Pseudomonas aeruginosa* and *Acinetobacter* sp. (Table 3). Vancomycin inhibitory activity on MRSA strains was above 90% on both tested concentrations (10 and 100 μg/ml), and bellow 30% against the VRE strain. Iminipen only inhibited the growth of ESBL-producing *E*.*coli*, which is the only Gram-negative bacterium sensitive to this drug. KPC, *P*. *aeruginosa* and *A*. *calcoaceticcus-baumannii* Cplx express carbapenes resistance, but are susceptible to polymyxin B.

**Table 3:** Inhibition of multi-resistant bacteria strains by Polydim-I and commercial antibiotics, in percentage ± SEM. Experimental details as described for Table 2. Three independent experiments were performed in triplicate.

		**GPC**			**GNB**	**GNBNF**	
**Drug/peptide(MW)**	Concentration (μg/ml) / μM	MRSA	VRE	*E*.*coli* ESBL	KPC	*P*. *aeruginosa*	*Acinetobacter* sp.
**Polydim-I**(2441.7 [M+H]^+^)	1 / 0.41	97.6 ±0.1	98.5 ±11.6	100.2 ± 0.3	97.8 ± 1.3	100.9 ±0.3	87.4 ± 1.7
	5 / 2.05	99.2 ±0.8	99.4 ±11,5	100,0 ± 0,1	102,7 ± 1,2	100,9 ± 1,1	100,1 ± 0,6
	30 / 12.3	101.3 ±0.2	98.5 ±11.6	99.7 ± 0.1	104.2 ± 0.2	100.3 ± 0.9	100.7 ± 0.2
**Meropenem** (383.5 g/mol)	10 / 26.1	NT	NT	93.0 ± 0.2	0.7 ± 0.07	1.8 ± 0.4	0.9 ± 0.05
	100 / 260.1	NT	NT	98.6 ± 0.5	0.8 ± 0.02	0.5 ± 0.1	1.4 ± 0.4
**Polymyxin B** (1204 g/mol)	10 / 8.3	NT	NT	100.5 ± 0.5	99.0 ± 0.7	99.8 ± 0.0	88.5 ± 1.2
	100 / 83	NT	NT	99.8 ± 0.05	100.0 ± 0.5	99.4 ± 0.5	98.3 ± 1.3
**Vancomycin** (1449 g/mol)	10 / 6.9	90.7 ± 0.05	26.5 ± 0.8	NT	NT	NT	NT
	100 / 69	95.4 ± 0.4	29.0 ± 0.2	NT	NT	NT	NT

**GPC:** gram-positive cocci. **GNB:** gram-negative bacteria. **GNBNF:** gram-negative bacteria non-spore forming. **NT:** non tested because there is no indication for use.

The results of the evaluation of the Polydim-I inhibitory activities against the strains expressing multi-resistant genes are shown in Table 3. Polydim-I presented a potent inhibition of the multidrug resistant bacteria growth, inhibiting 100% of all strains at concentration of 1μg/ml (0.41μM) except for *Acinetobacter* sp. that presented a MIC_100_ of 5μg/ml (2.05μM, Table 3). The MIC_50_ for the peptide against the multi-resistant bacteria would be bellow 1μg/ml concentration, and were not calculated. Polydim-I is much more efficient against ESKAPE pathogens than the ubiquitous drugs employed.

### 3.2 Secondary structure evaluation

CD and ATR-FTIR spectroscopy were used to investigate Polydim-I secondary structure in the aqueous environment and that induced by the presence of membrane mimetic systems. SDS solutions above the critical micelle concentration and asolectin LUVs present anionic character, a common feature of bacterial membranes [15]. Solutions were buffered at pH 5.5, mimicking the acidic environment of infected tissues and making His residues protonated.

**Fig 1:**
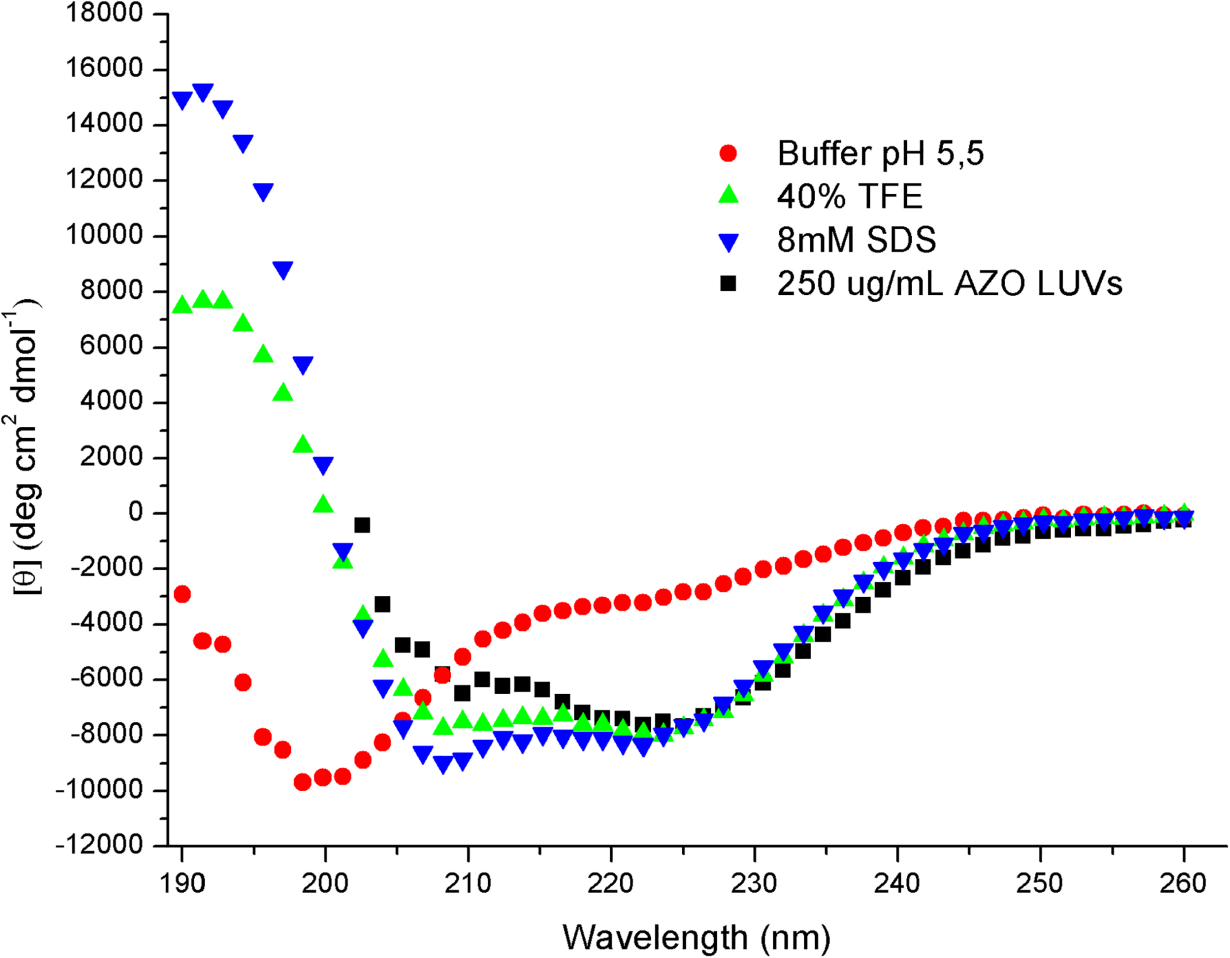
CD spectra of Polydim-I, obtained at 20 μM concentration, at 25^°^C, in different environments. Spectra are the average of 10 accumulations obtained at 50 nm/min. No smooth has been applied.

CD spectrum (Fig 1) of Polydim-I in buffer suggests an unordered structure and the peptide spectra in 40% TFE and in 8 mM SDS solutions exhibit the features of α-helical conformations with double minima around 208 and 223 nm. In the presence of anionic asolectin vesicles, the spectrum shows some deviation of the helical features, which could indicate the contribution of aggregated peptides. Nevertheless, the spectra do not indicate a preferential interaction with anionic environments as already found with other antimicrobial peptides [34,35,36].

**Fig 2:**
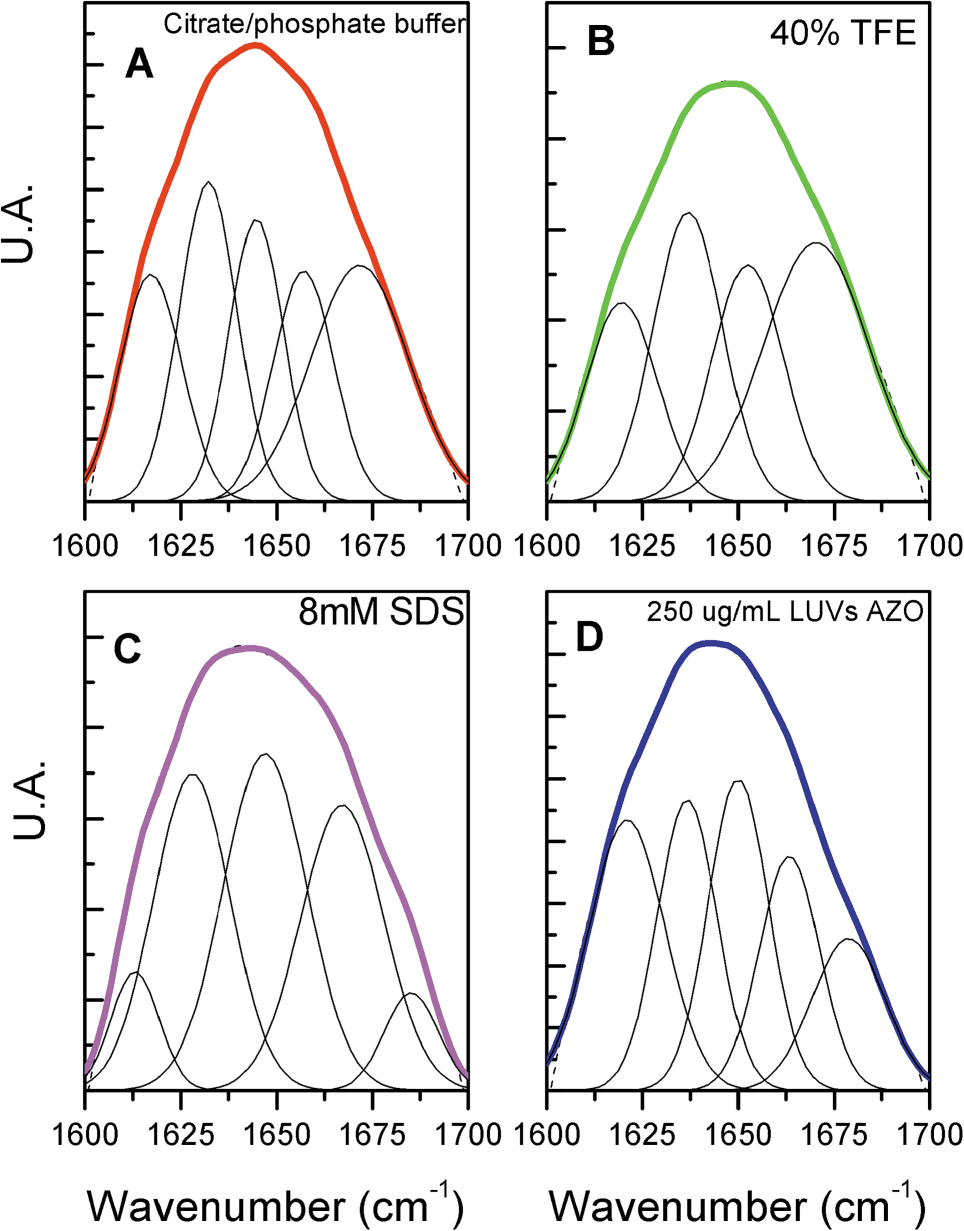
Deconvolution of the amide I band (1600–1700 cm^-1^) of the FTIR spectra of Polydim-I in different deuterated environments. The component peaks were obtained from the second derivatives (not shown) and result from a Gaussian curve fitting. The dashed lines are the experimental FTIR spectra (averaged over 20 scans) after the 13-data point Savitzky-Golay smoothing. The colored lines correspond to the sum of the components. The grey lines represent the deconvolution of the spectra in the different wave numbers. Spectra were acquired at 1 mM Polydim-I, at 25^°^C.

Fig 2 shows the spectra of the amide I region in the different environments, which superimpose the sum of the fitted components (100%). The deconvolution identifies the structural contributions at the respective wavenumbers. At 1620 cm^-1^, the aggregated forms, at 1635 cm^-1^, the β-sheet, at 1645 cm^-1^ the random structures, at1654 cm^-1^, the α-helix, and above 1665 cm^-1^, turns. In buffer, turns and random coil prevail, although some aggregated forms and β-sheet could be detected. These forms could result from peptide-peptide salt bridges and/or hydrophobic interactions. In TFE solution, known as a helical inductor environment, α-helix structures appeared at the expenses of turns and random coil reduction. In the presence of SDS micelles, the aggregated forms contributions significantly decrease with a proportional increase of the α-helix content. Although AZO LUVs exhibit an anionic character, it is mostly made of zwitterionic phospholipids and Polydim-I spectra in this environment hold similarities with the TFE solution spectra, however with an increased content of aggregated forms. The β-sheet content remained constant in the different media.

Table 4 compares the contributions of the secondary structure elements as obtained from CD and FTIR experiments. This latter technique allows an estimative of the aggregated content of peptide. In buffer, β-sheet, turns and aggregation could be due to electrostatic attraction between positively and negatively charged residues, either intra- or inter-chains. These structures contribute somewhat differently in the deconvolution of CD and IR spectra. The presence of the helix inductor TFE, and the membrane mimetics, SDS solution and AZO vesicles suspension, induce significant contents of helical and β-sheet structures. In the presence of the strong anionic environment of SDS micelles, we find the lowest aggregation content and highest ordered structure content. Low aggregation is an important feature to maximize antimicrobial activity and the higher anionic character of SDS when compared to asolectin LUVs suggests the relevance of the stronger electrostatic interactions, which can also be found in Gram-negative and Gram-positive bacteria [15].

**Table 4:** Secondary structure elements (%) of Polydim-I as determined by the deconvolution of CD and FTIR spectra.

Secondary structure evaluation (%)								
	Buffer, pH 5.5		40% TFE		8 mM SDS		250 μg/mL AZO LUV	
	*CD*	*IR*	*CD*	*IR*	*CD*	*IR*	*CD*	*IR*
**α-helix**	8	-	25	21	29	32	24	23
**β-sheet**	32	21	23	25	22	28	21	21
**turns**	23	45	23	36	22	34	25	31
**aggregated**	ND	16	ND	18	ND	6	ND	25
**random coil**	37	18	30	-	28	-	31	-

ND, not determined; -, absent

We also observed that Polydim-I structure should be of low amphipathicity, considering the calculated hydrophobic moment of 0.109 [37]. Amphipathicity has been often related to the activity of antimicrobial peptides on membranes, however as postulated by Wimley (2010) [38] it is not necessary for peptides to exhibit a perfect amphipathicity for improved biological activity and specificity.

### 3.3 Zeta-potential determinations

**Fig 3:**
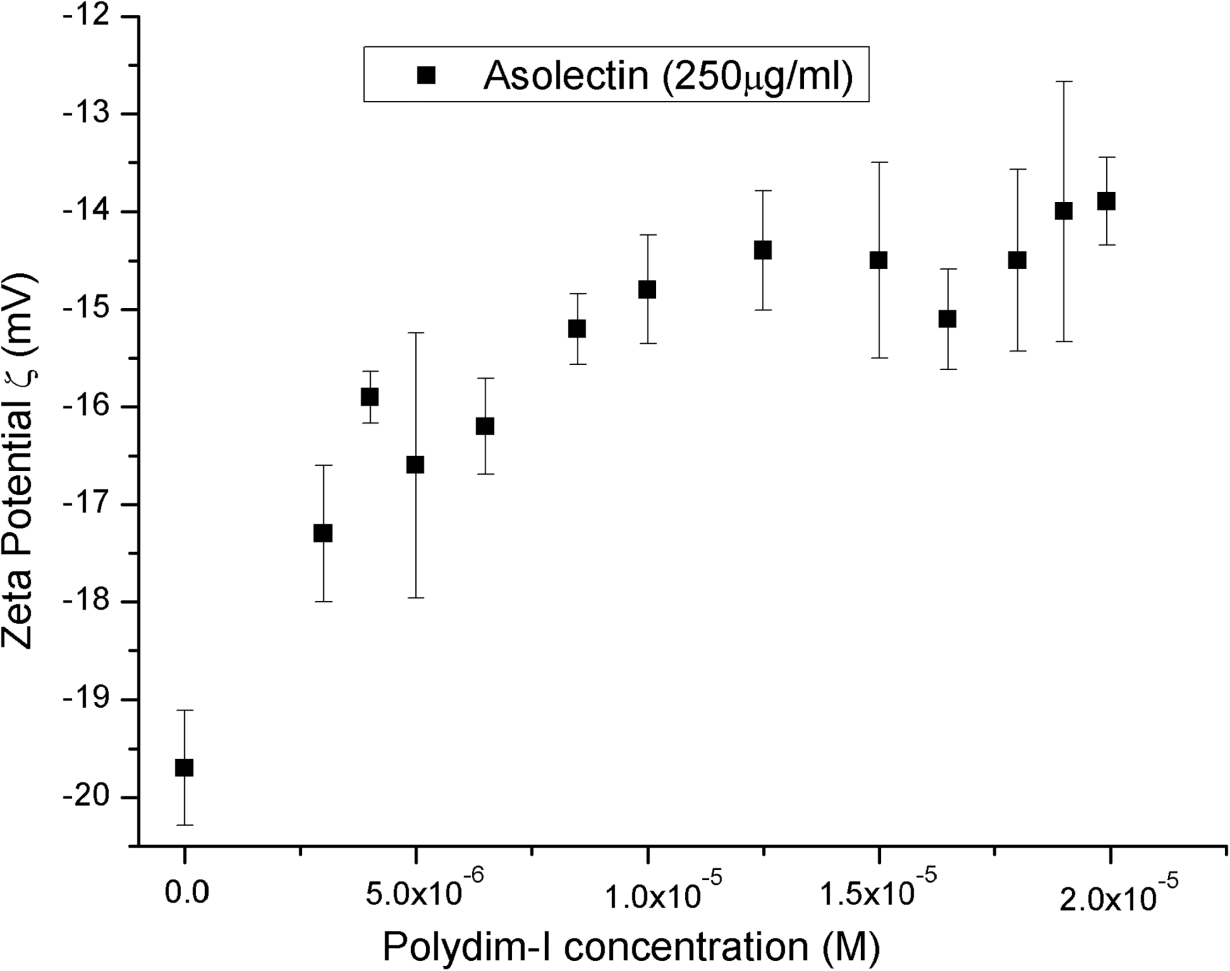
Zeta-potential isotherm obtained at 25^°^C, in the absence (ζ_0_) and presence of 250 μg/μL AZO vesicles. A series of buffered peptide solutions, pH 5.5, at increasing concentrations were mixed with an aliquot of AZO vesicles suspension and left for 30 minutes to equilibrate. Error bars represent the standard deviation of 3 measurements.

Considering that Polydim-I might target the phospholipid matrix of cell membranes as other antimicrobial peptides do, the binding to and accumulation on the outer leaflet of vesicles are important steps for membrane destabilization, before pore formation or the occurrence of any bilayer destabilization. These events can be assessed through zeta potential measurements. Zeta-potential values reflect the remaining charge density which were neither neutralized nor screened. Changes in zeta potential are used to quantify the extension of the interaction between peptides and phospholipid vesicles and changes in size are related to peptides inducing vesicles aggregation or acting in the detergent mode [39]. In the absence of peptide, AZO vesicles are characterized by an average zeta-potential value of -19.7 ± 0.6 mV (ζ_0_). Fig 3 shows that the interaction with the positively charged peptide causes the vesicles charging up as the peptide concentration increases. The variation of vesicle size does not indicate strong aggregation or micellization and it ranged from 115 to 179 nm throughout the experiment. Polydim-I gradually increases zeta-potential, tending to a plateau at concentrations above 10 μM. This finding either indicates poor binding or peptide aggregation on the vesicle surface. Considering the results from the FTIR experiments that indicated around 31% peptide aggregation in AZO vesicles (Table 4), this latter possibility seems to be the case.

### 3.4 Lipid bilayers electrical measurements

**Fig 4:**
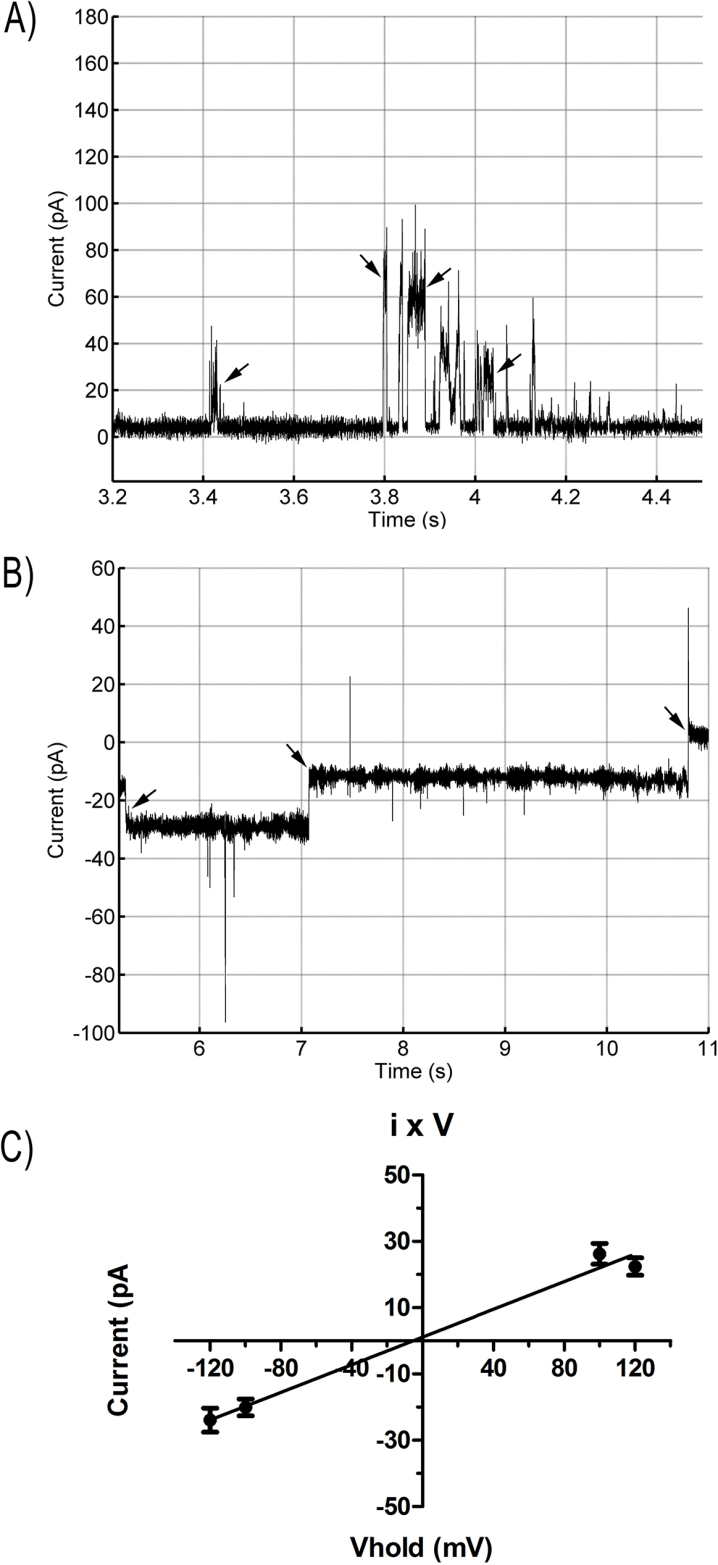
Representative recordings of Polydim-I (0.7 to 2 μM) single channel incorporation in AZO bilayers using a Nanion Port-a-Patch device and PatchControl software. Solution: 150 mM HCl (symmetrical). Arrows indicate some channel apertures or closings. A) V_hold_ = +100mV, Pore conductances = 265 and 629 pS; B) V_hold_ = -100mV, Pore conductance = 161 pS; C) Representative current-voltage linear relation of the pores formed by Polydim-I peptide in AZO bilayers (R^2^ = 0,9850; slope of 0.2088). Measurements of the single channels currents and duration of apertures were performed using PatchMaster software. Other analyses were performed in GraphPad Prism 5.0 Software. Six independent experiments were performed.

The peptide Polydim-I was able to induce channel-like activity in lipid bilayers (LBs) of asolectin at concentrations ranging from 0.7 to 2 μM (n = 6, independent experiments), using a symmetrical 150 mM HCl solution, within 10 min incubation time. Higher concentrations of the peptide (> 5μM) induced fast (2–3 seconds) LB breakdowns after applying our standard initial protocol (V_hold_ = -100 mV). Pore formation was observed under positive and negative voltage pulses (Fig 4A and 4B), and the current *versus* voltage relation (I x V) showed no rectification. In fact, the I x V regression resulted in a straight line passing near 0 (R^2^ = 0.9850), with constant slope of 0.2088, indicating that the pores formed by Polydim-I in the lipid bilayer are ohmic conductors, i.e., the pore resistance does not change with the applied voltage (V_hold_) (Fig 4C). Average unitary channel conductance was calculated under different V_hold_ pulses of -120, -100, +100 and +120 mV, and they are presented in Table 5, although no significant statistical differences were found between these conditions (non-parametric one-way ANOVA followed by Dunn’s multiple comparison test). The highest conductance opening was observed at the V_hold_ of -100 mV, reaching 2159 pS. Pores of different conductance levels were observed (Fig 4, Table 5), suggesting the organization of peptide monomers to form larger channel-like structures. P_open_ and dwell time values were estimated for V_hold_ of -100 and +100 mV under constant stimulus protocol, and are shown in Table 5. Dwell times were very short and similar for both conditions (280 and 320 ms respectively; unpaired t-test did not detect difference between the two conditions), while P_open_ was higher at +100 mV pulse (9.5%) than at -100mV (5.5%).

**Table 5:** Polydim-I induced pores in anionic (AZO) bilayers according to the applied voltage (V_hold_). Mean (± SEM), minimum and maximum conductance of pores obtained in 3–6 different experiments for each V_hold_ condition. Dwell time (time duration of channel aperture) and P_open_ of pores (probability of pore aperture during a recording, calculated by the sum of time of all apertures under a V_hold_ divided by the total time of recording in that same V_hold_ condition) averaged after 3 experiments. Measurements of the single channels currents and duration of apertures were performed using PatchMaster software. Other analyses were performed in GraphPad Prism 5.0 software.

**V**_**hold**_ **(mV)**	**Conductance (pS)**	**Minimum conductance (pS)**	**Maximum conductance (pS)**	**Dwell time (ms)**	**P**_**open**_ **(%)**
**-120mV**	211.4 ± 29.4	20.8	843.3		
**-100mV**	291.6±26.9	18.0	2159.0	280±140	5.5
**+100mV**	256.6±18.2	31.0	944.0	320±110	9.5
**+120mV**	209.6±25.0	41.7	483.3		

## 4 Discussion

The peptide Polydim-I was isolated from the venom of the social wasp *Polybia dimorpha* from the Brazilian savanna. This peptide presented *in vitro* and *in vivo* activities against the rapid growth of mycobacteria *Mycobacterium abscessus* subsp. *massiliense* [31] that is known to cause infectious diseases in humans in hospital environments after intramuscular injections and other small invasive procedures [31,40]. The *M*. *abscessus* subsp. *massiliense* presented cell wall disruptions after the treatment with Polydim-I, as verified by scanning electron microscopy (SEM), possibly due to the pore formation. The antibiotic clarithromycin, an inhibitor of protein synthesis, didn’t alter the morphology of the mycobacteria [31].

Another specie of social wasp from the same genus, *P*. *paulista*, also presents AMPs with strong activity against standard ATCC microbes, evaluated with the same strains used in the present work [41,42]. Polydim-I was active against standard ATCC strains, with lower MIC_50_ against *S*. *aureus* and *E*. *coli*. Other strains, *E*. *faecalis*, *P*. *aeruginosa*, *A*. *calcoaceticus baumannii* were less sensitive to the peptide (Table 1). This may be due to a less permeable membrane of these later strains that endow the microorganism with some intrinsic resistance and could have reduced the peptide permeability [43–45]. Besides LPS presence in gram-negative bacteria, membrane lipid composition varies among different bacteria species and strains, and the lipid composition of membranes has been referred as crucial to the activity of lipid targeting antimicrobials [10,24,46,47].

Polydim-I activity against standard ATCC strains is lower than that presented by conventional antibiotics (Tables 1 and 2). The only response similar to a reference drug (Vancomycin) was the MIC_50_ against *S*. *aureus*. The difference in the response of the bacteria strains with no resistance mechanisms is that the conventional antibiotics can easily reach and bind to their targets [48]. Since in these strains there is no inactivation or elimination of the antimicrobial agent, all the molecules transported into the cell will exert their effect on the molecular targets, successfully eliminating the microorganism [49].

The phenotype of the multi-resistant microorganisms tested in this study differs from the standard strains by the ability to produce extended spectra beta lactamases and carbapenemases. These are present at high concentrations in the periplasmic space, and inactivate the beta-lactam antibiotics by ring hydrolysis. The main mechanism of Gram-positive bacteria resistance is characterized by the changes in the receptor for the antimicrobial molecules, generally located on the plasma membrane or in the cell wall [6,48,50,51].

The control experiments with the multi-resistant strains and the standard drugs attested the good accuracy and sensitivity of the assays. The multi-resistant bacteria are far more sensitive to this peptide than the ATCC strains. MIC_100_ was very low, and it was not possible to calculate MIC_50_ due to the strong activity of the peptide (Table 3). These results indicated a higher and more specific response of the multidrug resistant pathogens to Polydim-I in comparison with the standard strains.

The Gram-negative multi-resistant bacteria were inhibited by peptide concentrations lower than polymixyn B and meropenem, which in the presence of different resistance mechanisms are the only therapeutic choices. Gram-negative bacteria can express one or more resistance mechanisms, conferring resistance against antibiotics such as cephalosporins, aminoglycosides, quinolones and other classes of antimicrobials [52,53]. Bacterial infections caused by multidrug resistant pathogens have become more frequent, making the empiric therapeutic choice a true challenge [54].

The results with Gram-positive multi-resistant bacteria were also very promising since Polydim-I inhibited 98% of the growth of MRSA and VRE strains at the concentration of 1μg/mL. Vancomicin inhibited up to 95% of the growth of MRSA at 10 and 100μg/mL while in the same concentrations growth inhibition of VRE was only 26.5–29% due to the resistance of this strain to this antibiotic.

Polydim-I was described by das Neves et al (2016) as a new class of AMP peptide [31]. We can expect some differences in the activity due to its novel sequence when comparing with other wasp peptides such as mastoparans. For instance, unlike most mastoparans, Polydim-I does not affect mammal cells, but presented selective toxicity towards bacteria [31]. Selective toxicity of AMPs towards bacteria is predominantly due to its interaction with the lipid bilayer, and the differences of composition between mammal cells and bacteria, that confer anionic character to the latter [55]. In the present manuscript we found that Polydim-I was more effective against ESKAPE bacteria when compared to ATCC antibiotic sensitive strains. Nevertheless, some AMPs have shown to be more active against MDR strains of bacteria when compared to susceptible ones. Vila-Farres and co-workers (2012) compared the MICs of 15 different AMPs and found that HNP-1 (Defensin Human Neutrophil Peptide-1) showed better activity against colistin-resistant *A*. *baumannii* (3.25 mg/L) when compared to the MIC for colistin susceptible *A*. *baumannii*, which was 15 times higher (50 mg/L). Mastoparan form *Vespula lewisi* presented a MIC four times lower for the resistant strain, only 1 mg/L, when compared to the sensitive one [56]. Mastoparan, as Polydim-I, causes abnormalities in the cell surface of bacteria [57].

Reasons for the stronger activity of Polydim-I against ESKAPE pathogens could be attributed to the differences on lipid composition of bacteria presenting mechanisms of resistance and sensitive ones [24,58], which have been compared since the 1970’s. Changes in net charge, fluidity and thickness of the lipid bilayer can be expected together with the lipid composition alterations caused by the expression of some of the known mechanisms of resistance [59]. Modifications in the lipid compositions of resistant bacteria might change the interaction of the AMPs with the lipid matrix [55,56,59]. According to Lohner (2009), qualitative and quantitative differences of membrane lipid compositions among microbes, and the changes that occur due to the expression of resistance mechanisms is an important goal of lipidomics, which will give support to a better understanding of membrane-AMP interactions [55].

A probable, but not exclusive, mechanism of action of Polydim-1 is the destabilization of the bacterial membrane by interaction with the lipid bilayer, in the same manner as other AMPs, inducing the free flow of water and solutes inwards and outwards the cells, leading to lysis. Therefore, the mechanisms of resistance, such as the production of inactivating enzymes, cannot affect the peptide action on the bacterial membrane. Polydim-I was effective 30 days after infection induced in mice in a previous study, reinforcing the hypothesis that no mechanism of resistance was induced during the experiment [31]. Since das Neves [31] raised the hypothesis of possible pore formation, we further investigated the peptide structure and interaction with mimetic membranes to confirm the occurrence of that mechanism.

Polydim-I, with +2.5 net charge and mean hydrophobicity, 0.080, calculated according to Eisenberg et al., 1984 [37], was found to exhibit helical and β-sheet structures at approximately the same content, and it may aggregate in the absence of stronger electrostatic interactions (Fig 2 and Table 4). This feature could be part of its selective performance. It binds to negatively charged vesicles, as indicated in the zeta potential experiments, partially neutralizing its charges (Fig 3). Polydim-I induced the disruption of the mycobacteria cell wall, as observed by scanning electron microscopy, but did not present toxicity as no macrophage membranes damage was observed [31]. Together these characteristics, low cytotoxicity and tendency to aggregate in environments with low anionic character, as are mammalian membranes, suggest that the selectivity of Polydim-I towards microbial membranes is related to differences in the electrostatic interaction with membranes that leads or not to aggregation of the peptide. The selective behavior of other wasp venom peptides from *Polybia* species was also correlated to important electrostatic interactions [60].

In our experiments with artificial lipid bilayers, Polydim-I presented channel-like activity as shown by the electrical measurements (Table 5 and Fig 4). Low concentration of the peptide was crucial to obtain stable electrical pore recordings in the artificial bilayers. The pore conductance under the applied V_hold_ presented no significant differences between positive and negative stimuli. The pores showed an ohmic I x V relation, and therefore the channel resistance does not change with the voltage applied in the bilayer. The pores formed by Polydim-I remain open for short periods of time (about 300ms), and have a higher probability to open under positive voltage pulses.

The formation of conductive pores was previously described for other peptides isolated from solitary and social wasp venoms, such as anoplin [61], HR-1 [39], eumenine mastoparan-EF and–ER [34], and eumenitin, eumenitin-F and eumenitin-R [34,62]. Some of these wasp peptides formed rectified pores, whose conductance (or resistance) changes according to the V_hold_ (see [34] Rangel et al., 2011).

Polydim-I linked two compartments isolated by a lipid bilayer, allowing ions to pass through. Ion movement across the formed pores result in measurable currents that were analyzed as conductance of those pores. The aim of this experiment was to show that the direct interaction of the peptide with membrane lipids can be responsible, at least, for ionic unbalance in the bacteria. Furthermore, pore formation in artificial lipid bilayers also indicates that the peptide does not necessarily require a protein receptor in the membrane to interact with it. It goes directly to the lipid matrix. Polydim-I formed pores of different conductance levels, suggesting that several peptide molecules (and/or even clusters) organize to form larger pores, with 100 times higher conductance than the average minimum observed. Lipid bilayer breakdown usually occurred a few seconds after larger pores incorporation. These observations also corroborate the findings that indicated some peptide aggregation.

In our results the evidence of Polydim-I aggregation was found in four different experiments: ATR-FTIR, CD spectroscopy, zeta potential determination and electrical measurement of lipid bilayers. We have observed that MIC_50_ was 5 to 100 times lower than MIC_100_ for most of the ATCC sensitive bacteria strain. Besides the differences of lipid bilayer compositions among bacteria used in our study and the electrostatic peptide interactions, the lower antimicrobial effect of Polydim-I in these ATCC strains at higher concentrations might be due to the increasing aggregation in that condition [15]).

## 5 Conclusions

Despite of great advances in medicine, the continuously acquired domain over many diseases is systematically threatened by the raising of severe infections’ incidence, caused by bacteria with unprecedented adaptation and dissemination capabilities. In the present study, the wasp peptide Polydim-I presented antimicrobial activity against standard bacteria strains deprived of mechanisms of resistance, and against multi-resistant strains. Furthermore, the inhibition of bacterial growth was more potent when the peptide was tested against strains expressing elaborated mechanisms of drug resistance. Polydim-I was more potent on inhibiting the growth of multi-resistant bacteria than commercial antibiotics. Our results also showed the interactions of this peptide with mimetic membranes, indicating that at least one of the modes of action would be pore formation in the bacterial membrane that would minimize the development of resistance mechanisms as a membrane receptor is not necessarily required [55]. This set of experiments also suggests that the selective mode of action of Polydim-I towards bacterial cells is possibly related to strong electrostatic interactions.

The significant difference of activity of the peptide against ESKAPE pathogens when compared to ATCC strains indicates that studies on the differences of the membrane lipid composition between these bacteria could be important to the design of new drugs. Polydim-I activity against many MDR pathogens seems to be an additional quality of this peptide, as it already showed to be safe to mice in *in vivo* assays and against mammal cells *in vitro*, and effective on both experimental models [31]. Polydim-I has a potential use as antimicrobial drug because it has broad spectrum against MDR microbial, regardless their mechanism of resistance. Moreover, Polydim-I could be used as a protective layer in materials to avoid biofilm formation.

As pointed out by Mingeot-Leclercq and Décout [59] it is a major challenge “to shift from a ‘hit’ scaffold to a ‘lead compound’”. This molecule can lead to the development of antibacterial drugs against bacteria strains that causes high morbidity index in patients with infectious diseases.

## Supporting information

S1 Table Raw data of inhibition rates of antibiotics and Polydim-I against ATCC bacteria. (PDF)

S2 Table Raw data of inhibition rates of antibiotics and Polydim-I against MDR strains. (PDF)

S3 Table Raw data from the CD spectra acquisition for Polydim-I in the different environments. (XLSX)

S4 Table Raw data from the FTIR spectra acquisition for Polydim-I in the different environments. (XLSX)

S5 Table Raw data from the zeta potential experiments for Polydim-I in the presence of AZO LUVs. (XLSX)

S6 Table Data that originated Fig 4C graph. (PDF)

S7 Table Data from bilayer recordings used in Table 5. (PDF)

S1 File Data points from representative bilayer recording shown in Fig 4A. (TXT)

S2 File Data points from representative bilayer recording shown in Fig 4B. (TXT)
