# Supplementary material for: Polydim-I antimicrobial activity against MDR bacteria and its model membrane interaction
Source: PLoS One. 2017 Jun 1;12(6):e0178785. doi: 10.1371/journal.pone.0178785 (PMC5453574; doi:10.1371/journal.pone.0178785)
Supplement: S1 Table — (PDF) [file pone.0178785.s001.pdf]

S 1 Table –Inhibition rates of antibiotics (A) and Polydim-I (B) against ATCC bacteria.

A)

Table format: XY		X	A			B			C			D			E		
		Antibiotic (μg/ml)	S. aureus ATCC			E. faecalis ATCC			E. coli ATCC			P. aeruginosa ATCC			A. calcoaceticcus ATCC		
		X	Mean	SD	N	Mean	SD	N	Mean	SD	N	Mean	SD	N	Mean	SD	N
1	Vancomycin	10	90.95	1.35	3	74.60	2.30	3									
2	Vancomycin	100	95.20	0.70	3	89.75	0.45	3									
3	Meropenem	10							97.50	1.70	3	94.95	4.85	3	99.05	0.65	3
4	Meropenem	100							96.15	0.05	3	96.75	5.35	3	100.15	0.85	3
5	Polymyxin B	10							87.60	6.70	3	98.90	0.10	3	92.55	1.75	3
6	Polymyxin B	100							85.35	5.35	3	97.90	2.00	3	99.80	0.10	3

B)

Table format: XY		X	A		B		C		D		E	
		Polydim-I (μg/ml)	S. aureus ATCC		E. faecalis ATCC		E. coli ATCC		P. aeruginosa ATCC		A. calcoaceticcus ATCC	
		X	A:Y1	A:Y2	B:Y1	B:Y2	C:Y1	C:Y2	D:Y1	D:Y2	E:Y1	E:Y2
1	Title	400	86.67	92.30	92.66	91.10	88.59	86.68	52.14	48.46	70.35	69.03
2	Title	200	73.88	87.89	86.93	84.80	84.86	86.53	40.73	45.53	58.49	57.65
3	Title	150	71.38	72.53	54.32	70.00	81.27	82.85	37.29	42.29	52.30	57.64
4	Title	100	67.83	61.65	47.84	46.14	66.29	67.28	28.32	37.81	48.17	48.38
5	Title	50	64.26	59.17	45.18	40.72	43.91	45.23	23.70	36.55	45.75	41.92
6	Title	30	56.66	58.15	29.81	24.00	37.74	21.45	22.95	32.65	41.11	34.38
7	Title	5	42.93	54.78	13.48	17.12	34.07	17.85	20.10	29.08	22.07	28.49
8	Title	1	40.87	53.19	2.76	13.43	20.82	-29.89	18.58	28.88	20.44	22.17
