# Supplementary material for: Polydim-I antimicrobial activity against MDR bacteria and its model membrane interaction
Source: PLoS One. 2017 Jun 1;12(6):e0178785. doi: 10.1371/journal.pone.0178785 (PMC5453574; doi:10.1371/journal.pone.0178785)
Supplement: S2 Table — (PDF) [file pone.0178785.s002.pdf]

S 2 Table –Inhibition rates of antibiotics (A) and Polydim-I (B) against MDR bacteria.

A)

Table format: XY		X	A			B			C				D		E			F		
		Antibiotic (µg/ml)	MRSA			VRE			E.coli ESBL				KPC		P. aeruginosa			Acinetobacter sp.		
		X	Mean	SD	N	Mean	SD	N	Mean	SD	N	Mean	SD	N	Mean	SD	N	Mean	SD	N
1	Vancomycin	10			3			3												
2	Vancomycin	100			3			3												
3	Meropenem	10	90.75	0.05	3	26.55	0.85	3	93.00	0.20	3	0.74	0.07	3	1.85	0.45	3	0.89	0.05	3
4	Meropenem	100	95.45	0.45	3	29.00	0.20	3	98.65	0.65	3	0.85	0.03	3	0.50	0.10	3	1.45	0.45	3
5	Polymyxin B	10							100.45	0.55	3	99.05	0.75	3	99.80	0.00	3	88.50	1.20	3
6	Polymyxin B	100							99.85	0.05	3	100.45	0.55	3	99.44	0.54	3	98.35	1.35	3

B)

Table format: XY		X	A		B		C		D		E		F	
		Polydim-I (µg/ml)	MRSA		VRE		E.coli ESBL		KPC		P. aeruginosa		Acinetobacter sp.	
		X	A:Y1	A:Y2	B:Y1	B:Y2	C:Y1	C:Y2	D:Y1	D:Y2	E:Y1	E:Y2	F:Y1	F:Y2
1	Title	400	106.24	104.68	108.01	104.07	100.39	99.78	110.44	108.74	102.07	101.16	103.74	102.95
2	Title	200	105.19	104.95	122.08	95.25	100.29	99.25	108.51	107.43	100.72	101.40	104.08	102.62
3	Title	150	104.78	103.98	120.14	91.36	98.99	100.39	105.32	108.82	99.66	99.99	101.69	102.06
4	Title	100	102.75	102.01	118.69	91.09	99.01	99.23	105.38	105.59	99.38	99.71	101.42	102.45
5	Title	50	101.79	101.21	117.83	92.58	100.38	99.61	105.78	104.41	100.94	99.62	101.14	101.51
6	Title	30	101.46	101.09	116.29	89.19	99.81	99.57	104.41	103.98	99.41	101.13	100.45	100.91
7	Title	5	99.99	98.37	110.95	87.87	100.49	100.39	101.55	103.91	99.78	102.07	99.49	100.76
8	Title	1	97.76	97.53	110.14	86.88	100.46	99.93	96.56	99.13	100.59	101.28	85.62	89.10
