# Supplementary material for: Polydim-I antimicrobial activity against MDR bacteria and its model membrane interaction
Source: PLoS One. 2017 Jun 1;12(6):e0178785. doi: 10.1371/journal.pone.0178785 (PMC5453574; doi:10.1371/journal.pone.0178785)
Supplement: S6 Table — (PDF) [file pone.0178785.s006.pdf]

S6_Table – Data that originated Fig. 4C graph.

Polydim-I i x v data sheet

	A	B	C	D
	-120mV	-100mV	100mV	120mV
	Y	Y	Y	Y
1	-9.9	-98.1	8.6	5.0
2	-16.0	-30.8	7.4	8.6
3	-21.0	-64.8	29.0	14.8
4	-22.3	-14.2	9.5	23.4
5	-32.1	-34.1	11.1	25.9
6	-27.2	-23.4	12.3	35.8
7	-33.3	-6.2	29.6	29.6
8	-35.8	-8.7	10.7	24.6
9	-34.6	-9.5	20.2	38.3
10	-34.7	-37.9	4.5	40.7
11	-35.8	-12.2	10.5	34.6
12	-96.3	-12.6	16.7	42.0
13	-32.7	-10.6	12.9	14.8
14	-12.4	-12.3	11.7	6.8
15	-16.1	-11.1	13.6	14.2
16	-29.0	-9.9	13.6	29.3
17	-10.2	-8.7	11.7	8.9
18	-20.1	-10.5	11.7	7.0
19	-9.3	-9.9	13.6	9.9
20	-18.5	-8.3	10.4	16.0
21	-11.1	-9.8	15.4	38.2
22	-4.3	-10.5	16.7	24.1
23	-18.2	-5.3	16.7	
24	-25.6	-4.6	9.8	
25	-22.5	-10.5	13.0	
26	-6.4	-5.9	16.0	
27	-52.6	-6.8	10.5	
28	-6.2	-9.6	14.2	
29	-3.1	-8.0	30.2	
30	-2.5	-11.7	34.0	
31	-3.7	-15.4	19.7	
32	-101.2	-12.3	16.7	
33	-15.4	-14.8	37.6	
34	-13.6	-9.2	17.2	
35	-17.3	-16.1	8.6	
36	-9.9	-9.9	18.5	
37		-23.5	22.8	
38		-18.5	18.5	
39		-15.8	29.6	
40		-22.8	12.9	
41		-5.0	50.0	
42		-91.3	39.5	
43		-8.6	45.7	
44		-38.8	93.8	
45		-25.9	93.1	
46		-33.9	94.4	
47		-13.0	27.8	
48		-20.1	25.9	
49		-10.2	17.8	
50		-9.3	19.1	
51		-18.5	40.1	
52		-15.4	20.4	
53		-32.1	62.9	
54		-36.4	87.6	
55		-88.3	76.5	
56		-58.6		
57		-25.9		
58		-6.6		
59		-24.7		
60		-46.9		
61		-6.4		
62		-3.5		
63		-8.7		
64		-3.5		
65		-14.6		
66		-4.5		

Polydim-I i x v row stats

Row stats		X	A		
		X Title	1		
		X	Mean	SEM	N
1	-120mV	-120	-23.914	3.636	36
2	-100mV	-100	-20.083	2.504	66
3	100mV	100	26.227	3.130	55
4	120mV	120	22.386	2.634	22

Polydim-I i x v linear regression

Linear reg.		A
		1
		Y
1	Best-fit values	
2	Slope	0.2088 ± 0.01820
3	Y-intercept when X=0.0	1.154 ± 2.010
4	X-intercept when Y=0.0	-5.529
5	1/slope	4.790
6	95% Confidence Intervals	
7	Slope	0.1304 to 0.2871
8	Y-intercept when X=0.0	-7.496 to 9.805
9	X-intercept when Y=0.0	-51.20 to 38.34
10	Goodness of Fit	
11	r^2	0.9850
12	Sy.x	4.021
13	Is slope significantly non-zero?	
14	F	131.5
15	DFn, DFd	1.000, 2.000
16	P value	0.0075
17	Deviation from zero?	Significant
18	Data	
19	Number of X values	4
20	Maximum number of Y replicates	1
21	Total number of values	4
22	Number of missing values	0
