# Supplementary material for: Polydim-I antimicrobial activity against MDR bacteria and its model membrane interaction
Source: PLoS One. 2017 Jun 1;12(6):e0178785. doi: 10.1371/journal.pone.0178785 (PMC5453574; doi:10.1371/journal.pone.0178785)
Supplement: S7 Table — (PDF) [file pone.0178785.s007.pdf]

S7_Table – Data from bilayer recordings used in Table 5.

Polydim-I pore conductance data sheet

	A	B	C	D
	-120 mV	-100 mV	100 mV	120 mV
	Y	Y	Y	Y
1	20.83	981.00	31.00	41.67
2	25.83	18.00	45.00	56.67
3	30.83	31.00	46.00	58.33
4	35.83	35.00	52.00	71.67
5	51.67	35.00	55.00	74.17
6	53.33	37.00	68.00	82.50
7	77.50	40.00	68.00	118.33
8	82.50	45.00	74.00	123.33
9	82.50	46.00	80.00	123.33
10	85.00	49.00	80.00	133.33
11	92.50	50.00	86.00	190.00
12	103.33	52.00	86.00	195.00
13	113.33	53.00	86.00	200.83
14	128.33	59.00	89.00	205.00
15	133.33	62.00	89.00	215.83
16	134.17	62.00	90.00	244.17
17	144.17	64.00	92.00	246.67
18	151.67	65.00	95.00	288.33
19	154.17	66.00	98.00	298.33
20	167.50	68.00	99.00	318.33
21	169.17	68.00	99.00	319.17
22	175.00	74.00	102.00	339.17
23	185.83	74.00	104.00	350.00
24	187.50	74.00	105.00	462.50
25	213.33	74.00	105.00	483.33
26	226.67	74.00	105.00	
27	241.67	77.00	107.00	
28	267.50	77.00	111.00	
29	272.50	80.00	117.00	
30	277.50	80.00	117.00	
31	288.33	83.00	117.00	
32	289.17	83.00	117.00	
33	298.33	86.00	117.00	
34	298.33	87.00	120.00	
35	431.67	87.00	123.00	
36	438.33	87.00	123.00	
37	467.50	90.00	123.00	
38	802.50	92.00	127.00	
39	843.33	92.00	129.00	
40		93.00	129.00	
41		93.00	130.00	
42		95.00	133.00	
43		96.00	136.00	
44		98.00	136.00	
45		99.00	136.00	
46		99.00	139.00	
47		99.00	142.00	
48		99.00	142.00	
49		99.00	142.00	
50		99.00	142.00	
51		102.00	154.00	
52		105.00	157.00	
53		105.00	160.00	
54		105.00	167.00	
55		105.00	167.00	
56		105.00	167.00	
57		105.00	167.00	
58		106.00	170.00	
59		111.00	172.00	
60		111.00	173.00	
61		111.00	178.00	
62		111.00	179.00	
63		117.00	179.00	
64		122.00	179.00	
65		123.00	181.00	
66		123.00	185.00	
67		125.00	185.00	
68		126.00	191.00	
69		130.00	194.00	
70		136.00	197.00	
71		136.00	202.00	
72		142.00	204.00	
73		146.00	210.00	
74		148.00	216.00	
75		148.00	228.00	
76		148.00	231.00	
77		151.00	234.00	
78		154.00	234.00	
79		154.00	238.00	
80		154.00	247.00	
81		154.00	247.00	
82		158.00	247.00	
83		161.00	250.00	
84		173.00	253.00	
85		176.00	259.00	
86		182.00	259.00	
87		185.00	271.00	
88		185.00	272.00	
89		185.00	278.00	
90		188.00	284.00	
91		191.00	284.00	
92		197.00	290.00	
93		197.00	296.00	
94		201.00	296.00	
95		201.00	302.00	
96		210.00	302.00	
97		228.00	309.00	
98		231.00	321.00	
99		234.00	327.00	
100		234.00	327.00	
101		235.00	340.00	
102		247.00	345.00	
103		247.00	352.00	
104		247.00	370.00	
105		247.00	376.00	
106		253.00	383.00	
107		259.00	395.00	
108		259.00	401.00	
109		277.00	420.00	
110		278.00	457.00	
111		296.00	475.00	
112		308.00	493.00	
113		321.00	500.00	
114		327.00	536.00	
115		339.00	542.00	
116		339.00	556.00	
117		341.00	568.00	
118		357.00	592.00	
119		357.00	629.00	
120		364.00	697.00	
121		379.00	741.00	
122		382.00	765.00	
123		388.00	806.00	
124		407.00	814.00	
125		407.00	876.00	
126		432.00	931.00	
127		432.00	938.00	
128		445.00	944.00	
129		469.00		
130		469.00		
131		475.00		
132		481.00		
133		505.00		
134		506.00		
135		518.00		
136		533.00		
137		568.00		
138		586.00		
139		623.00		
140		648.00		
141		734.00		
142		784.00		
143		883.00		
144		901.00		
145		913.00		
146		913.00		
147		926.00		
148		938.00		
149		950.00		
150		1037.00		
151		1222.00		
152		1259.00		
153		1284.00		
154		1358.00		
155		1517.00		
156		2159.00		

Polydim-I pore dwell time data sheet

	A	B
	-100 mV	100 mV
	Y	Y
1	0.069	0.190
2	0.045	1.745
3	0.074	0.068
4	0.076	0.095
5	0.049	0.745
6	0.035	2.223
7	0.044	0.134
8	0.040	0.202
9	0.115	0.073
10	0.297	0.159
11	0.153	0.130
12	0.226	0.067
13	0.089	0.483
14	0.101	0.074
15	3.258	0.123
16	0.227	0.186
17	0.341	0.123
18	0.163	0.100
19	0.632	0.090
20	0.053	0.140
21	0.274	0.102
22	0.018	0.190
23	0.058	0.044
24		0.251

Polydim-I pore Popen

Vhold	Dwell time (s)	n	EPM	sum open time (s)	rec time since open (s)	Popen (%)
-100mV	0.28	23	0.14	6.44	117.6	5.47
100mV	0.32	24	0.11	7.737	81.2	9.53
